# Effects of a standardized 40% ellagic acid pomegranate (*Punica granatum* L.) extract on seminiferous tubule histopathology, diameter, and epithelium thickness in albino Wistar rats after heat exposure

**DOI:** 10.14202/vetworld.2019.1261-1265

**Published:** 2019-08-17

**Authors:** Budi Utomo, Nurfitri Rahmah Daningtia, Gandul Atik Yuliani, Wiwik Misaco Yuniarti

**Affiliations:** 1 Department of Reproduction Veterinary, Faculty of Veterinary Medicine, Universitas Airlangga. Jl. Mulyorejo, Kampus C Unair, Surabaya 60115, Indonesia; 2 Faculty of Veterinary Medicine, Universitas Airlangga. Jl. Mulyorejo, Kampus C Unair, Surabaya 60115, Indonesia; 3 Department of Basic Veterinary Science, Faculty of Veterinary Medicine, Universitas Airlangga. Jl. Mulyorejo, Kampus C Unair, Surabaya 60115, Indonesia; 4 Department of Veterinary Clinics, Faculty of Veterinary Medicine, Universitas Airlangga. Jl. Mulyorejo, Kampus C Unair, Surabaya 60115, Indonesia

**Keywords:** diameter, epithelium thickness, heat, pomegranate extract, seminiferous tubule

## Abstract

**Background and Aim:**

It has long been known that the spermatogenic tissue is very sensitive to temperatures higher than its physiologic temperature and causing cessation of activity and resulting in sterility. This study investigated the effect of a standardized 40% ellagic acid extract of pomegranate on the histopathology, diameter, and epithelial thickness of seminiferous tubules in albino rats exposed to heat.

**Materials and Methods:**

Twenty-five male albino Wistar rats were randomized at 7-8 months of age to five treatment groups. Group C was not treated; Group T0 was treated with 0.5% of Na carboxymethyl cellulose (CMC) 2 ml/day and exposed to heat. T1, T2, and T3 were treated with 75, 150, and 300 mg/kg/day of a standardized 40% ellagic acid extract of pomegranate (*Punica granatum* L.), respectively. The animals were orally administered Na CMC or pomegranate extract and were exposed to sunlight for 15 min at 40°C-42°C for 14 days. The animals were sacrificed on day 15 and the testes were removed for histological evaluation and measurement of seminiferous tubule diameter and epithelium thickness.

**Results:**

The diameter of seminiferous tubules from rats exposed to heat and treated with 300 mg/kg/day pomegranate extract was larger and the epithelia thicker than those in the other groups (p<0.05). The protective effects of the standardized 40% ellagic acid extract may have been mediated by its antioxidant activity.

**Conclusion:**

Compared with controls, administration of 300 mg/kg/day of a standardized 40% ellagic acid extract of *P. granatum* L. for 14 days increased seminiferous tubule diameter and epithelium thickness in albino Wistar rats exposed to heat.

## Introduction

Male animals are considered infertile if conception does not occur after mating for 12 months or more. Infertility may be caused by decreased spermatozoa production or motility that reduces the number that reaches the ovum and is able to penetrate the egg membrane during fertilization [1]. Male reproductive function begins with spermatogenesis, the development of spermatozoa from spermatogonia, which is regulated by testosterone, follicle-stimulating hormone (FSH), and luteinizing hormone produced by Leydig and Sertoli cells in the seminiferous tubules of the testes [2]. Fertility depends on both the quality of spermatozoa the successful delivery of spermatozoa to ovum. In addition to hormones, spermatogenesis is influenced by factors that inhibit epididymal function, radiation, and increased temperature [1]. Heat exposure causes an imbalance of the production of free radicals and antioxidants, with an increase in free radicals resulting in oxidative stress [3].

Pomegranate (*Punica granatum* L.) is an ornamental plant that is rarely cultivated in Indonesia, where its potential benefits for treating male fertility are not appreciated. Pomegranate is a source of plant antioxidants high in polyphenols, anthocyanins, and Vitamin C that can prevent damage caused by oxidative stress and protects cell from free radicals [4]. Vitamin C has antioxidant activity that is synergistic with polyphenol antioxidants present in pomegranates such as anthocyanins, punicalagin, ellagic acid, and gallic acid in protecting cells from free radical damage. Pomegranates can maintain fertility by protecting seminiferous tubule cells from free radical damage [5]. Vitamin C deficiency can result in testicular tissue damage and reduction in the number of spermatogenic cells in seminiferous tubules, with consequent decreases in seminiferous tubule diameter, thickness of the seminiferous tubule epithelium, and testicle weight [6].

This study investigated the effect of a standardized 40% ellagic acid pomegranate (*P. granatum* L.) extract on seminiferous tubule diameter and thickness in albino Wistar rats after exposure to heat stress.

## Materials and Methods

### Ethical approval

The study was conducted following ethical guidelines for the use of experimental animals and as approved by the ethics committee of the Faculty of Veterinary Medicine, Universitas Airlangga, Indonesia.

### Time and place

This study was conducted from June to December 2017 at Pharmacology Laboratory, Faculty of Medicine and Veterinary Pathology Laboratory, and Faculty of Veterinary Medicine, Universitas Airlangga, Surabaya.

### Materials

Powdered pomegranate extract (*P. granatum* L.) was standardized as 40% ellagic acid solution (Xi’an Bio-Technology Co. Ltd., Xi’an, China). Other reagents included hematoxylin and eosin stains, physiological saline, 30%, 50%, 70%, 80%, 95%, and 100%, ethanol, 5% Na carboxymethyl cellulose (CMC), 10% buffered formalin, xylol, paraffin, glycerin, balsam, ether, animal feed, and drinking water. The tools used in this study include electric scales, spoons, pharmacy paper, label paper, plastic, Erlenmeyer flasks, Petri dishes, stirrers, food pipes, cage cleaning tools, masks, gloves, food and drink containers, surgical instruments (tweezers, scalpel, blade, and scissors), object-glass, pipettes, hot plates, coverslips, and a light microscope (Nikon Eclipse E 100 LED) were used.

### Experimental procedures

Rats were weighed at the beginning and the end of experimental procedures and were sacrificed by exposure to ether. Testes and seminiferous tubule were dissected, weighed, cleared of adhering connective tissue, and assayed immediately. The testes were weighed and the seminiferous tubule weight, length, and thickness were measured. The diameter and thickness of seminiferous tubule from five rats per treatment were measured using an ocular micrometer in a light microscope (Nikon Eclipse E-100) and the average size of seminiferous tubule and germinal cell layer thickness was calculated. Seminiferous tubule diameter (µm) was measured by ocular micrometer and results were given as average. One of testis from each rat was fixed in 10% formalin for histopathological examination. About 25 white male Wistar rats randomly allocated at 7-8 months of age to five study groups. Group C was not treated. Group T0 was treated with 0.5% of Na CMC 2 ml/day, Group T1 was treated with 75 mg/kg/day pomegranate extract, Group T2 was treated with 150 mg/kg/day, and Group T3 was treated with 300 mg/kg/day pomegranate extract. The animals in Groups T0-T3 were exposed to temperatures of 40°C-42°C produced by exposure to 15 min of sunlight. The animals were treated for 14 days.

### Histopathological examination

Testis and seminiferous tubule tissue were fixed in 10% buffered formalin for 24 h. Serial tissue sections were processed by standard histological techniques and stained with for observation by light microscopy with a Nikon Eclipse E-100 Microscope. Five randomly selected fields of view in each slide of the histopathological tissue preparations were scored at 100×.

### Statistical analysis

The statistical analysis was performed by SPSS 20.0 software (IBM, USA). The results were presented as means value (±SD). One-way analysis of variance and *post hoc* analysis with Duncan’s test for multiple comparisons was used to compare each group and data among the groups, respectively. p<0.05 was considered statistically significant.

## Results and Discussion

**Figure-1:**
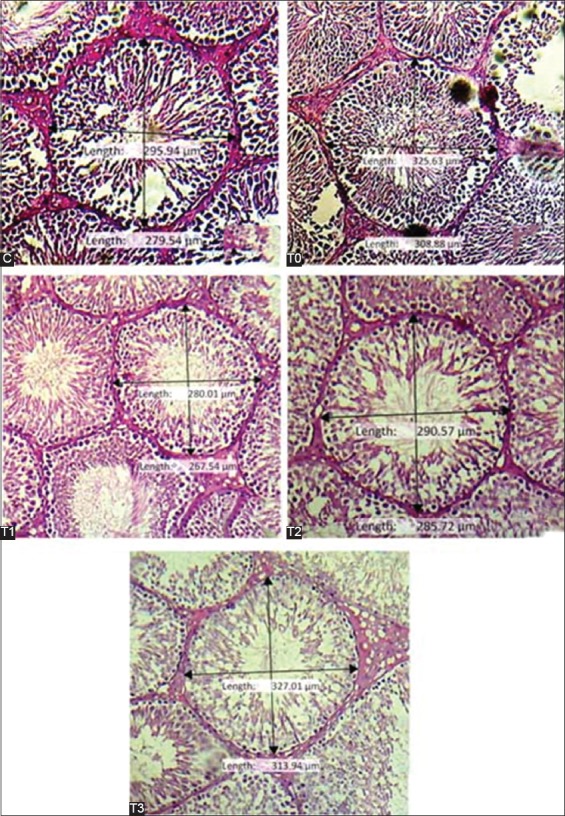
Histopathology and diameter of seminiferous tubules from Groups C, T0, T1, T2, and T3 (100×, hematoxylin-eosin staining).

**Figure-2:**
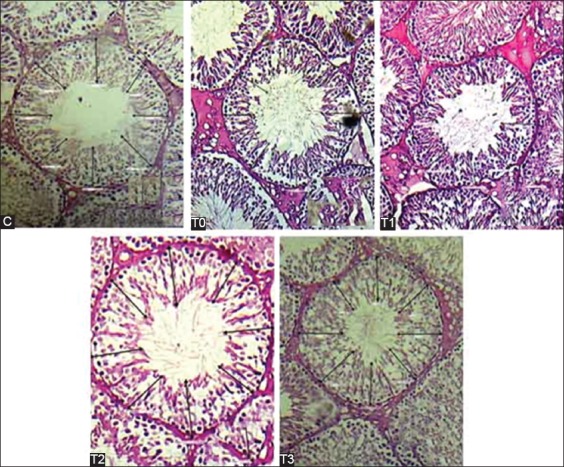
Histopathology and epithelial thickness of seminiferous tubules in Groups C, T0, T1, T2, and T3 (100×, hematoxylin-eosin staining).

Testis tissue from control Group (C) rats had a normal microscopic structure. The diameter of the seminiferous tubules in Group T0 rats was significantly different from that in Groups C, T1, T2, and T3 rats (p<0.05). The diameters of Group C and Group T1 were not significantly different (p>0.05), but both were different from the diameters of the tubules in Groups T0, T2, and T3 (p<0.05). The diameters of the tubules in Group T2 and Group T3 were significantly different. The smallest mean seminiferous tubule diameter (261.85±15.85 µm) was seen in Group T0 group and the largest (335.93±25.85 µm) in Group T3 (Figure-1 and Table-1). The thickness of the seminiferous tubule epithelium in Group C, which was not treated, and that in Group T2, which was exposed to heat and 150 mg.kg/day pomegranate extract, was not significantly different. The difference in the tubule epithelium thickness in Group T1 (heat and 75 µm/kg/day extract) and Group T2 (heat and 150 mg/kg/day) was not significantly different (p>0.05). The seminiferous tubular epithelium in T3 was the thickest compared with the control group and the other treatments (p<0.05, Figure-2 and Table-1). Heat exposure decreased the thickness of the seminiferous tubule epithelium, and the change was primarily caused by a loss of germ cells. Spermatogenic cells in the seminiferous tubule epithelium and Leydig cells in interstitial areas were markedly prominent in Group T3 rats. The reduction of the seminiferous tubule diameter in the Group T0 rats indicates that heat caused atrophy of the tubules and led to morphological changes and disturbed spermatogenesis in the testis. The results are consistent with a previous report of a positive relationship between seminiferous tubules diameter and spermatogenesis activity [7].

**Table 1:** Seminiferous tubule diameter and epithelium thickness.

Treatment group	Diameter, µm (mean±SD)	Epithelium thickness, µm (mean±SD)
C (no treatment)	261.85^b^±15.85	86.27^b^±10.44
T0 (Na CMC 0.5/day)	221.43^a^±10.50	78.11^a^±14.24
T1 (75 mg/kg/day)	253.59^b^±18.73	80.24^a^±11.69
T2 (150 mg/kg/day)	300.17^c^±29.84	87.75^b^±14.99
T3 (75 mg/kg/day)	335.93^d^±25.85	95.9^c^±8.49

Superscripts in the same column indicate significant differences following by Duncan’s MCT (p<0.05). SD=Standard deviation

The study showed that the treatment of the rats within Groups T1, T2, and T3 with pomegranate extract significantly increased the diameter of the seminiferous tubules and the thickness of the tubule epithelium compared with Group T0 rats treated with heat and Na CMC. The effects were probably the result of the antioxidant properties of the pomegranate extract. In another study, exposure to cigarette smoke led to reductions in seminiferous tubule diameter and epithelial thickness that were inhibited by honey supplementation. It was suggested that the protective effect was mediated by the antioxidant activity of honey [8]. Cyclophosphamide-induced reduction in the spermiogenesis index, tubule differentiation index, serum testosterone, and testis weight has been found to be partially returned to normal by an extract of *Crataegus monogyna* with known antioxidant activity [9].

Treatment with the pomegranate extract resulted in significant increases in seminiferous tubule diameter compared with the Groups C and T0. The largest increase was seen in Group T3, which was given 300 mg.kg/day for 14 days. The standardized 40% ellagic acid extract of *P. granatum* L. has antioxidant activity. Antioxidants stabilize free radicals by satisfying electron deficiencies and inhibiting the chain reaction of free radical formation that causes cell damage during heat exposure. Administration of the standardized pomegranate extract at 300 mg/kg may have suppressed free radical formation. The resulting prevention of the damage of cell membranes and preservation of hormone receptors involved in the promotion of spermatogenesis has been shown to result in an increase in spermatogenic cells [10]. Increase in the spermatogenic and Sertoli cells in the tubular epithelium results in narrowing of seminiferous tubule lumen, thickening of seminiferous epithelium, and increase the diameter of the seminiferous tubule [11]. According to Franca and Russell [12], there is a positive relation between tubular diameter and the spermatogenic activity. The treatment with all of the concentrations of pomegranate increased tubular diameter and thickness of the epithelium. The results were differing significantly from the control group, suggesting that there was improvement with pomegranate given to the treatment groups. The dominant antioxidant compound contained in *P. granatum* L. extract is anthocyanin, which is likely to influence hormonal regulation of FSH, luteinizing hormone and testosterone, thereby affecting the growth and development of and Sertoli cells and spermatogonia [13,14].

A significant increase in spermatogenic cells in Group T3 compared with Groups C and T0 resulted in corresponding significant increase in the thickness of the seminiferous tubule epithelium and diameter. The increases were consistent with anthocyanin reduction of oxidative stress caused by heat exposure and maintenance of spermatogenic cells by inhibition of enzyme damage to maintain normal spermatogenesis. Increased epithelium thickness has been associated with increased numbers of Sertoli and spermatogenic cells [15]. The antioxidant activity of anthocyanin has been attributed to the donation of a single electron to oxidant molecules and thus increasing the number of spermatozoa [16].

The decrease in seminiferous tubule epithelium thickness in the T0 comparison group resulted from loss of Sertoli and spermatogenic cells or spermatogenesis inhibition [17] as well as the possibility of germinal cell apoptosis. Apoptosis may occur spontaneously in seminiferous epithelium or as a response to various factors including chemotherapy agents, high temperature, and hormone activity [18]. The study results show that 300 mg/kg/day of the pomegranate extract promoted increases in the seminiferous tubule diameter and epithelial thickness, leading to increased efficiency of spermatogenesis.

## Conclusion

A standardized 40% ellagic acid extract of pomegranate (*P. granatum* L.) at a dose of 300 mg/kg/day increased seminiferous tubule diameter and epithelium thickness in Wistar albino rats following exposure to heat.

## Authors’ Contributions

BU and NRD carried out the main study procedures, WMY performed the histopathological analysis of the testis, and GAY performed the statistical analysis. All authors have read and approved the final version of the manuscript.
